# Zwitterionic Surfactant Modified Acetylene Black Paste Electrode for Highly Facile and Sensitive Determination of Tetrabromobisphenol A

**DOI:** 10.3390/s16091539

**Published:** 2016-09-21

**Authors:** Xiaoyun Wei, Qiang Zhao, Weixiang Wu, Tong Zhou, Shunli Jiang, Yeqing Tong, Qing Lu

**Affiliations:** 1 Key Laboratory of Environment and Health, Ministry of Education & Ministry of Environmental Protection, and State Key Laboratory of Environmental Health, School of Public Health, Tongji Medical College, Huazhong University of Science and Technology, Wuhan 430030, China; m201475163@hust.edu.cn (X.W.); farutong@163.com (Q.Z.); sam_woo123@foxmail.com (W.W.); 15392896632@163.com (T.Z.); utopianjiang@163.com (S.J.); 2 Hubei Provincial Center for Disease Control and Prevention, Wuhan 430079, China

**Keywords:** tetrabromobisphenol A, acetylene black, zwitterionic surfactant, electrochemical determination

## Abstract

A electrochemical sensor for the highly sensitive detection of tetrabromobisphenol A (TBBPA) was fabricated based on acetylene black paste electrode (ABPE) modified with 3-(*N*,*N*-Dimethylpalmitylammonio) propanesulfonate (SB3-16) in this study. The peak current of TBBPA was significantly enhanced at SB3-16/ABPE compared with unmodified electrodes. To further improve the electrochemical performance of the modified electrode, corresponding experimental parameters such as the length of hydrophobic chains of zwitterionic surfactant, the concentration of SB3-16, pH value, and accumulation time were examined. The peak currents of TBBPA were found to be linearly correlated with its concentrations in the range of 1 nM to 1 µM, with a detection limit of 0.4 nM. Besides, a possible mechanism was also discussed, and the hydrophobic interaction between TBBPA and the surfactants was suggested to take a leading role in enhancing the responses. Finally, this sensor was successfully employed to detect TBBPA in water samples.

## 1. Introduction

Tetrabromobisphenol A (TBBPA) is a currently intensively used brominated flame retardant (BFR) in manufacturing for printing circuit boards, plastic polymers, and electronic equipment due to its excellent thermal stability and superior flame retardant efficiency [1]. Along with the heavy usage and wide spread application of TBBPA, environmental problems occurred when TBBPA products were disposed of inappropriately. In recent decades, TBBPA were reported to be found in various environmental samples around the world, including water [2], soil [3], air [4], dust [5], sewage sludge [6], as well as in biological samples [7]. It can also migrate into creatures through bioaccumulation due to its lipophilicity, environmental stability, and poor degradability [8]. These situations suggest that TBBPA pollution has become a potential threat to public health. Evidence from epidemiological and animal studies have indicated that TBBPA can induce a number of adverse effects including endocrine disrupting effects [9], neurotoxicity [10,11], immunotoxicity [12,13], hepatotoxicity [14], and nepatotoxicity [15]. Therefore, with the purpose of managing TBBPA, it is of great importance to establish sensitive and simple methods to detect TBBPA.

At present, TBBPA detection methods are mostly based on liquid chromatography-mass spectrometry [16], gas chromatography–mass spectrometry [17], high performance liquid chromatography-electro-spray tandem mass spectrometry [18], and electrochemical sensors [19,20]. Compared with these methods, the electrochemical method is preferred owing to merits including simple operation, high sensitivity, fast response, and low cost. However, poor electrochemical performance of TBBPA has restricted the application of electrochemical methods [21,22]. To settle this problem, some sensing material, which could enhance the electrochemical response of TBBPA, must be developed to establish electrochemical methods for the highly sensitive detection of TBBPA.

Acetylene black (AB), a carbon nano-material, is a special kind of carbon black. Owing to properties including large surface area, porous structure, and remarkable conductivity, it has been extensively used in the fabrication for electrochemical sensors [23,24]. For example, AB has been successfully employed for the construction of sensors to increase the detecting sensitivity of some substances, such as p-nitrophenol [25] and vanillin [26]. Hence, AB might be used to enhance the determining sensitivity of TBBPA.

Surfactants are a type of amphiphilic molecules, which can be absorbed to the electrode surface through the hydrophobic interaction from their long hydrophobic C–H chains. They can not only modify the properties of the electrode surface, but also greatly affect the electrochemical process [27]. It had also been reported that surfactants could remarkably increase the enrichment ability of electrochemical sensors to nonionic organic compounds, such as nonyl phenol [28], phenol [29], and bisphenol A [30]. Therefore, surfactants were introduced in this method to increase the sensitivity of the sensor.

In this study, 3-(*N*,*N*-Dimethylpalmitylammonio) propanesulfonate (SB3-16), a kind of zwitterionic surfactant, was introduced for the modification of acetylene black paste electrode (ABPE). The response signals of TBBPA at the modified electrode were remarkably improved due to the superior electrochemical properties of AB and the good adsorption capacity of SB3-16. The possible mechanism was also discussed. The present sensor was employed to detect TBBPA in pool water samples, with the results in accordance with those from HPLC.

## 2. Experimental Section

### 2.1. Reagents

3,3′,5,5′-Tetrabromobisphenol A (TBBPA, 99%) was purchased from Sigma-Aldrich (St Louis, MO, USA). Standard solution of TBBPA (1.0 × 10^−2^ M) was prepared by dissolving certain amounts of TBBPA in acetonitrile. Acetylene black was supplied by STREM Chemicals (Newburyport, MA, USA). Paraffin oil, graphite powder, phenol, resorcinol, hydroquinone, octyl phenol, nonyl phenol, 3-aminophenol, 4-nitrophenol, hexadecyltrimethylammonium bromide (CTAB), Brij 58, and sodium dodecyl sulfonate (SDS) were acquired from Sinopharm Chemical Reagent Co., Ltd. (Shanghai, China). 3-(*N*,*N*-Dimethyllaurylammonio) propanesulfonate (SB3-12), SB3-14, SB3-16, SB3-18 were acquired from Aladdin (Shanghai, China). 4,4′-Dihydroxydiphenylmethane (BPF), BPA, tetrabromobisphenol A diallyl ether (TBBPA-DAE), and tetrabromobisphenol A bis(hydroxyethyl) ether (TBBPA-BHE) were obtained from the Tokyo Chemical Industry (Shanghai, China). BPAF and 4,4′-Sulphonylbis (2,6-dibromophenol) (TBBPS) were acquired from Meryer Chemical Technology Co., Ltd. (Shanghai, China). 0.1 M phosphate buffer of different pH value was prepared by mixing standard solutions of Na_2_HPO_4_ and NaH_2_PO_4_. All reagents used were of analytical grade without further purification. All solutions were prepared using distilled water.

### 2.2. Apparatus

Electrochemical measurements were performed using a computer-controlled CHI 830C electrochemical workstation (Shanghai Chenghua Apparatus, Shanghai, China), which contained a saturated calomel electrode (SCE), a platinum electrode, a SB3-16 modified acetylene black paste electrode. Electrochemical impedance spectroscopy (EIS) was performed with a CHI660D electrochemical workstation (Shanghai Chenhua Apparatus, Shanghai, China). All pH measurements were conducted at a PB-20 pH meter (Sartorius AG, Goettingen, Germany). HPLC was performed on an Agilent model 1200 series with UV detector (Agilent Technologies, Santa Clara, CA, USA). Elite Hypersil ODS column (150 mm × 4.6 mm, I.D. 5 µm) was used as the analytical column and the mobile phase consisted of methanol and water (85:15, *v*/*v*) at a flow rate of 1.0 mL·min^−1^. The sample injection volume was 20 μL, and the determination for TBBPA was set at a wavelength of 212 nm. 

### 2.3. Preparation of the SB3-16/ABPE

The SB3-16/ABPE was prepared as follows: 500 mg of acetylene black powder and 80 µL of paraffin oil were blended in an agate mortar and ground thoroughly to form a homogeneous paste for 30 min by hand. A portion of the paste was packed into the cave of the electrode, followed by the polish process against weighting paper until the surface was smooth. The solution of 0.05 mM SB3-16 was prepared by dissolving it in distilled water through 20 min ultrasonic agitation. After that, 10 μL of the gained SB3-16 solution was dropped onto the surface of ABPE for 5 min and then the unabsorbed SB3-16 was removed with distilled water. For comparison, the same method was applied for the preparation of carbon paste electrode (CPE) made of graphite powder and CPE modified with SB3-16 (SB3-16/CPE).

### 2.4. Sample Preparation

The water samples were collected from an outdoor pool of the city. The samples were firstly filtered by 0.45 μm membrane, and then the obtained water samples were divided into two. One was used to prepare 0.1 M phosphate buffer (pH = 7.0) for electrochemical detection and the other was then filtered by 0.22 μm membrane and mixed with methanol (1:1, *v*/*v*) for HPLC analysis.

## 3. Results and Discussions

### 3.1. Characterization of Electrodes

**Figure 1:**
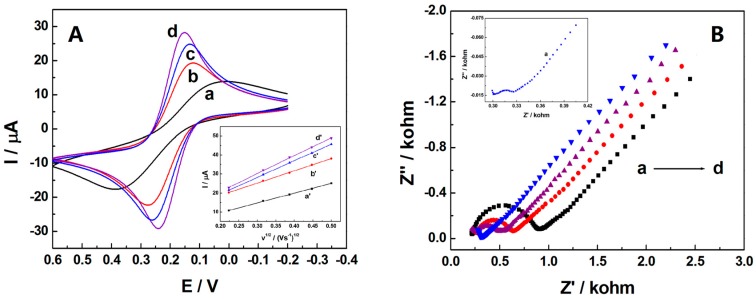
(**A**) Cyclic voltammograms of CPE (a), ABPE (b), SB3-16/CPE (c), and SB3-16/ABPE (d) in 0.1 M KCl solution containing 1.0 mM [Fe(CN)_6_]^4−/3−^ with scan rate of 100 mV·s^−1^. Inset: Plot of *I*_p_ versus *ν*^1/2^ (V·s^−1^)^1/2^ at CPE (a′), AB-CPE (b′), SB3-16/CPE (c’) and SB3-16/ABPE (d′); (**B**) Nyquist plots of SB3-16/ABPE (a), SB3-16/CPE (b), ABPE (c), and CPE (d) in 0.1 M KCl solution containing 5.0 mM [Fe(CN)_6_]^4−/3−^. Inset: The amplication of a.

Electrochemical performances of various working electrodes were investigated by cyclic voltammetry in 0.1 M KCl solution containing 1.0 mM [Fe(CN)_6_]^4−/3−^ and their cyclic voltammograms were shown in Figure 1A. A pair of redox peaks of [Fe(CN)_6_]^4−/3−^ were found at bare CPE (curve a), and the peak-to-peak separation (Δ*E*_p_) was 355 mV. While on ABPE (curve b), Δ*E*_p_ was 154 mV and the redox peak currents enhanced, which was possibly resulting from the remarkable electrical conductivity of AB. After modification with SB3-16, the electrochemical behavior of the [Fe(CN)_6_]^4−/3−^ on the electrodes was markedly increased, and the Δ*E*_p_ was reduced to 132 mV for SB3-16/CPE (curve c) and 89 mV for SB3-16/ABPE (curve d), indicating that SB3-16 could improve the hydrophobic property of electrodes, which might be helpful in the diffusion and adsorption of [Fe(CN)_6_]^4−/3−^ on the surface of CPE and ABPE. Inset of Figure 1 showed the linear relationship of peak currents and square root of scan rate for CPE (curve a′), ABPE (curve b′), SB3-16/CPE (curve c′), and SB3-16/ABPE (curve d′). On the basis of the Randles-Sevcik equation, shown below Equation (1) [31]:

*i*_p_ = (2.69 × 10^5^) *n*^3/2^*AD*^1/2^*ν*^1/2^*c* (1)

where *i*_p_ is the oxidation peak current (A), *c* is the concentration of [Fe(CN)_6_]^4−/3−^ (mol·cm^−3^), *ν* is the scan rate (V·s^−1^), *A* is the electroactive surface area (cm^2^), *n* is the electron transfer number, and *D* is the diffusion coefficient of the [Fe(CN)_6_]^4−/3−^ at electrode surface (cm^2^·s^−1^) (for [Fe(CN)_6_]^4−/3−^, *n* = 1, *D* = 6.7 × 10^6^). The electroactive surface area of ABPE, SB3-16/CPE and SB3-16/ABPE were calculated to be 0.0905 cm^2^, 0.122 cm^2^, and 0.135 cm^2^, respectively. According to the trace a in Figure 1A, the reaction process of CPE was more irreversible, and Equation (2):

*i*_p_ = (2.99 × 10^5^) *n* (α*n*)^1/2^*AD*^1/2^*ν*^1/2^*c* (2)

which is suitable for irreversible processes, was used to calculate electrochemical active surface area of CPE. α*n* was calculated to be 0.63 according to the slope of the plot of *E*_p_ versus ln *v*. Hence, the electroactive surface area of CPE was calculated to be 0.078 cm^2^. The results indicated that AB and SB3-16 could greatly improve the effective surface area of the electrodes.

Electrochemical impedance spectrum (EIS) was also applied to study the impedance changes of the electrodes after modification. The Nyquist plots were conducted in 0.1 M KCl solution containing 5.0 mM [Fe(CN)_6_]^4−/3−^ with frequency varied from 0.01 to 100,000 Hz. The corresponding Nyquist diagrams were shown in Figure 1B. For CPE (curve d), a large semicircle was shown in the high-frequency section, indicating a great electron transfer resistance. Compared with CPE, the semicircle of ABPE (curve c) was obviously smaller, suggesting AB could enhance the conductivity of the electrode and increase the electron transfer efficiency between the electrode and electrolyte. Moreover, the resistance decreased dramatically after the CPE (curve b) and ABPE (curve a) were modified with SB3-16 (curve a), indicating a smaller resistance to interfacial electron transfer. 

### 3.2. Cyclic Voltammetric Behaviors of TBBPA at the SB3-16/ABPE

**Figure 2:**
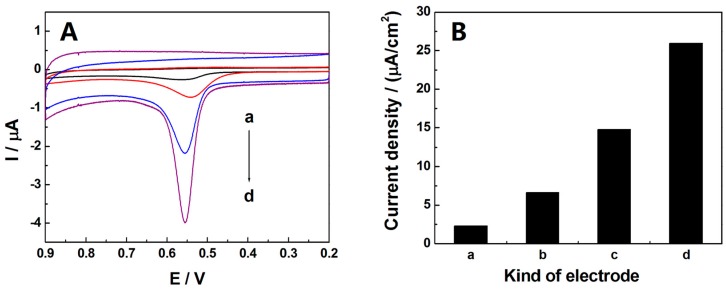
(**A**) Cyclic voltammograms of 0.5 µM TBBPA at the CPE (a), ABPE (b), SB3-16/CPE (c), and SB3-16/ABPE (d) in 0.1 M phosphate buffer (pH = 7.0) after a 240 s accumulation. Scan rate: 100 mV·s^−1^; (**B**) The current density derived from cyclic voltammograms responses of 0.5 µM TBBPA at the surface of different electrodes.

Cyclic voltammetric behaviors of 0.5 µM TBBPA in 0.1 M phosphate buffer (PBS, pH = 7.0) on various electrodes were investigated at a scan rate of 100 mV·s^−1^. As seen from Figure 2A, only one oxidation peak was shown at all electrodes from 0.2 to 0.9 V, suggesting the electrochemical process of TBBPA to be irreversible. As for CPE (curve a), the oxidation of TBBPA happened at the potential of 0.562 V and the current response was very poor. The peak current of TBBPA at ABPE was larger than that acquired at CPE with a negatively shifted peak potential of 0.541 V (curve b). These phenomena indicated that the oxidation of TBBPA was more prone to react at the ABPE, which might be ascribed to the superior electrochemical properties of AB. In contrast, when CPE (curve c) and ABPE (curve d) was modified with SB3-16, both of the oxidation currents of TBBPA increased rapidly. The significant increase of current response revealed that SB3-16 exhibited strong adsorption capacity towards TBBPA. Moreover, the highest oxidation peak current of TBBPA was occurred at SB3-16/ABPE, suggesting that the synergistic action might exist between AB and SB3-16. On the one hand, more SB3-16 could be adsorbed on the surface of ABPE because that ABPE possesses a higher surface area than CPE. On the other hand, SB3-16 might make the paraffin oil layer on the surface of ABPE become thinner though dissolving the paraffin oil, which could result in an exposition of the active sites of ABPE and increase the conductivity [32]. Furthermore, Figure 2B shows the current density derived from the cyclic voltammograms of 0.5 µM TBBPA at the surface of different electrodes and the results also demonstrate that the presence of AB and SB3-16 together lead to the increase of peak current of TBBPA. In conclusion, the SB3-16/ABPE could act as a very efficient promoter to improve the electrochemical behavior of TBBPA due to the synergistic effect of AB and SB3-16, and created the condition for sensitive detection of TBBPA.

### 3.3. The Possible Mechanism of Action of Surfactants

**Figure 3:**
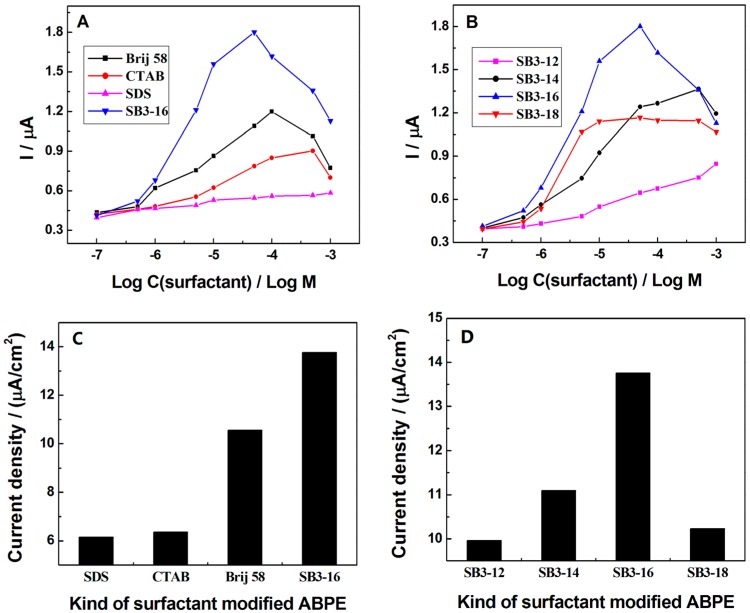
(**A**) The influences of different kinds of surfactants on the oxidation peak current of 0.5 µM TBBPA; (**B**) The effects of zwitterionic surfactant with different length of hydrophobic chains on the oxidation peak current of 0.5 µM TBBPA; (**C**) The current density of 0.5 µM TBBPA at the surface of different kind of surfactant modified ABPE; (**D**) The current density of 0.5 µM TBBPA at the surface of zwitterionic surfactant with different lengths of hydrophobic chain modified ABPE.

To explore the possible mechanism of action of surfactants, the influences of different surfactants—such as cationic surfactant cetyltrimethylammonium bromide (CTAB), zwitterionic surfactant SB3-16, anionic surfactant sodium dodecyl sulfonate (SDS), and neutral surfactant Brij 58—on the oxidation peak current of 0.5 µM TBBPA were studied. In Figure 3A, the oxidation peak currents of TBBPA were enhanced at all these surfactants (during a certain concentration range) modified ABPE. Surfactants could bind the substances through hydrophobic interaction and electrostatic interaction [33]. The oxidation peak current of TBBPA on zwitterionic surfactant SB3-16 and neutral surfactant Brij 58 modified electrodes were higher than that on cationic surfactant CTAB and anionic surfactant SDS modified electrodes. Therefore, it is supposed that the hydrophobic interaction might be critical in the binding TBBPA to the surfactants, rather than the electrostatic interaction. Moreover, the oxidation peak current of TBBPA at SB3-16/ABPE was the highest. It could be assumed that zwitterionic surfactant might act as an electronic conductor due to its own chemical structure (with both positive and negative charge), which could effectively facilitate the electrochemical oxidation of TBBPA [34]. Moreover, as shown in Figure 3C, the current density of 0.5 µM TBBPA at the surface of different surfactants modified ABPE also suggested that SB3-16 could enhance the electrochemical response of TBBPA more effectively. To further verify the above viewpoints, the effects of zwitterionic surfactant with different length of hydrophobic chains: SB3-12, SB3-14, SB3-16, and SB3-18 on the current response of TBBPA were also studied (Figure 3B). The peak current of TBBPA enhanced as the length of hydrophobic chains increasing from 12 to 16, indicating that the longer hydrophobic chains might possess stronger hydrophobic interactions to TBBPA, which was coordinated with the previous assumption. However, the peak current at SB3-18/ABPE was lower than that at SB3-16/ABPE. It was probably due to the fact that SB3-18 owns the longer hydrophobic chains, which would result in the longer distance for electron transfer from TBBPA to the electrode surface. The results of the effect of zwitterionic surfactant with different length of hydrophobic chains on the current density of 0.5 µM TBBPA (Figure 3D) were in consistent with above conclusions.

The concentration of SB3-16 also affected the oxidation peak current of TBBPA. Hu et al. has reported that different concentration of surfactant could form different shapes on the hydrophobic surface [35]. Therefore, a similar conclusion might be deduced to SB3-16. As shown in Figure 3A (curve SB3-16), the peak current first increased gradually during the SB3-16 concentration from 1 × 10^−7^ M to 1 × 10^−6^ M, suggesting that the SB3-16 might undergo monomer adsorption in this concentration. In the range of 1 × 10^−6^ M to 1 × 10^−5^ M, the peak current increased rapidly, which indicated that SB3-16 might form a loose monolayer on the surface of ABPE. When the concentration of SB3-16 was closed to its critical micelle concentration, the oxidation peak current of TBBPA tended to be stable. It was convinced that SB3-16 might form a compact monolayer and the density of this monolayer increased with SB3-16 concentration. However, as the concentration of SB3-16 continued to increase, the peak current consequently decreased, which might be caused by the micelle effect. Therefore, 5 × 10^−5^ M SB3-16 was employed in this study.

### 3.4. Electrochemical Process of TBBPA on SB3-16/ABPE

#### 3.4.1. Effect of Solution pH

**Figure 4:**
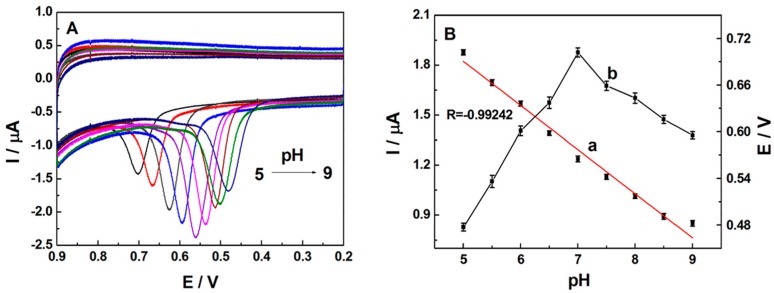
(**A**) Cyclic voltammograms of 0.5 µM TBBPA at SB3-16/ABPE in 0.1 M phosphate buffer of different pH: 5.0, 5.5, 6.0, 6.5, 7.0, 7.5, 8.0, 8.5, and 9.0; (**B**) The effects of pH on the peak current and potential. Scan rate: 100 mV·s^−1^.

In this study, 0.1 M PBS was used to study the effects of various pH on the electrochemical performance of electrode, with the concentration of TBBPA to be 0.5 µM (Figure 4). As shown in Figure 4B (curve a), as the PBS pH increased, the potential of oxidation peak shifted negatively. The peak potential (*E*_p_) was linear to pH value with a equation of *E*_p_(V) = −0.0563 pH + 0.9717 (R = 0.9924). Compared with the theoretical value of −0.059 V/pH, the slop value in our study was calculated to be −0.0563 V/pH, which might indicate that the number of electrons and protons that participated in the oxidation process were equal. As displayed in Figure 4B (curve b), the peak current value was observed to be maximum at the pH of 7.0 and then decreased with the further increase of pH. Therefore, the pH value of 7.0 was selected for further studies.

#### 3.4.2. Influence of Scan Rate

**Figure 5:**
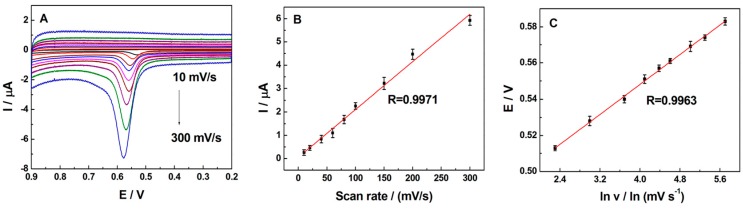
(**A**) Cyclic voltammograms of 0.5 µM TBBPA in 0.1 M phosphate buffer (pH = 7.0) on SB3-16/ABPE at dufferent scan rates: 10, 20, 40, 60, 80, 100, 150, 200, and 300 mV·s^−1^; (**B**) The linear relationship of peak current and scan rate; (**C**) The plot of peak potential (*E*_p_) and natural logarithm of scan rate (ln *ν*).

The impacts of scan rate (*ν*) on the electrochemical oxidation of 0.5 µM TBBPA at SB3-16/ABPE were explored in 0.1 M phosphate buffer (pH = 7.0). As shown in Figure 5A, the oxidation peak current of TBBPA was linear to the scan rate in the range of 10 mV·s^−1^ to 300 mV·s^−1^ (Figure 5B) and the equation was *i*_p_ = 0.0204*ν* + 0.06569 (R = 0.9971), revealing that the oxidation of TBBPA at the SB3-16/ABPE was a surface-controlled process. Similarly, E_p_ has a linear relation with Napierian logarithm of *ν* (ln *ν*) in the range of 10 mV·s^−1^ to 300 mV·s^−1^ (Figure 5C). The equation could be expressed as *E*_p_(V) = 0.02094 ln *ν* + 0.4745 (R = 0.9963). Regarding the totally irreversible electrode process and a surface-controlled (according to Laviron’s work [36]) *E*_p_ could be defined by the following Equation (3):

*E*_p_ = *E*° + R*T*/(α*nF*) ln[R*Tk*°/(α*nF*)] + R*T*/(α*nF*) ln *ν* (3)

where *E*° is the formal potential, α is the transfer coefficient, *T* is the temperature, *n* is the number of the electron transfer, *k*° is the electrochemical rate constant, *ν* is scan rate, and *F* is the Faraday constant. On the basis of above information, αn is calculated to be 1.23. Moreover, the method of Laviron’s half-peak widths was also used to calculate α*n*. According to this theory [37], *W*_1/2_ could be defined by the Equation (4):

*W*_1/2_ = 2.44R*T*/α*nF* (4)

Consequently, α*n* is calculated to be 1.16, which was in accordance with the above results. In general, α is set to be 0.5 in irreversible electrode process [38]. Therefore, the electron transfer number (*n*) was approximately 2 in this study. Since the number of electron was equal to that of proton, the electro-oxidation of TBBPA at SB3-16/ABPE was suggested to be a two-electron and two-proton process.

### 3.5. Influence of Accumulation Time

The influence of accumulation time on the oxidation peak current of 0.5 µM TBBPA was explored by cyclic voltammetry. The oxidation peak current enhanced when the accumulation time was below 240 s, and then the peak current reached a plateau as the accumulation time continued to increase. This phenomenon could be ascribed to the saturated adsorption of TBBPA on surface of SB3-16/ABPE. In our study, 240 s was chosen as the optimal accumulation time to ensure both of the sensitivity and efficiency of the sensor.

### 3.6. Analytical Properties

#### 3.6.1. Calibration and Limit of Detection

**Figure 6:**
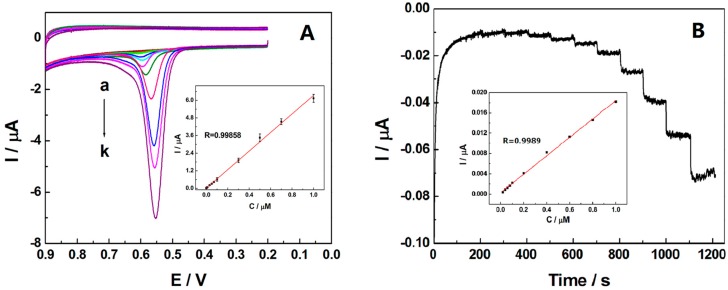
(**A**) Cyclic voltammograms of SB3-16/ABPE at various concentration of TBBPA, from a to k: 0.001, 0.005, 0.01, 0.03, 0.05, 0.07, 0.1, 0.3, 0.5, 0.7, and 1 µM in 0.1 M phosphate buffer (pH = 7.0), scan rate 100 mV·s^−1^. Inset: The plot of electrocatalytic peak current versus TBBPA concentration; (**B**) Amperometic current response of the SB3/ABPE by successive additon of TBBPA with concentration of 0.02, 0.04, 0.06, 0.08, 0.1 0.2, 0.4, 0.6, 0.8, and 1 µM into 0.1 M phosphate buffer (pH = 7.0). Applied potential: 0.7 V. Inset: The plot of electrocatalytic peak current versus TBBPA concentration.

Under the optimized conditions, the calibration curve for TBBPA was characterized by cyclic voltammetry. In Figure 6A, the oxidation peak currents increased linearly with the concentration of TBBPA ranging from 1 nM to 1 µM, with a linear regression of *I*_p_(µA) = 6.3197C(µM) + 0.0163 (R = 0.99858). The detection limit was calculated to be 0.4 nM at a ratio of signal to noise of 3 after 240 s accumulation. Additionally, the peak potential of the cyclic voltammetry shifts towards lower potentials as the concentration of TBBPA increases, which could be due to the fact that the diffusion of TBBPA to the electrode surface might become easier with the increase of TBBPA concentration. The comparison of this sensor with other published papers of TBBPA had been displayed in Table 1. The compared results indicated that the proposed method has the lower detection limit and wider linear range and could potentially be used to sensitively determine TBBPA. The higher sensitivity and wider linearity of this sensor could be ascribed to the combination of the superior electrochemical properties of AB and good hydrophobic adsorption of SB3-16 to TBBPA.

#### 3.6.2. Amperometric Response of SB3-16/ABPE

The amperometric current-time response of SB3-16/ABPE to TBBPA at a potential of 0.7 V was also studied in the 0.1 M phosphate buffer (pH = 7.0) with a step of a certain concentration of TBBPA added. As displayed in Figure 6B, the current response increased immediately and became steady within 3 s, indicating that the modified electrode exhibited an exceedingly rapid and sensitive response toward TBBPA. It was suggested in the inset that the current response of the sensor was in linear relation with the concentration of TBBPA ranging from 0.02 µM to 1 µM with the regression equation of *i*_p_(µA) = 0.0184C(µM) + 0.000327 (R = 0.998). The detect limit was calculated to be 6.4 nM, which was higher than the detect limit obtained from cyclic voltammetry (0.4 nM). It was probably due to the method theory of amperometry and cyclic voltammetry being different [38], and the experimental parameters of cyclic voltammetry were optimized in this study. More importantly, amperometric current-time curve was mainly to illustrate that this sensor might have a promising potential for on-site practical detections.

#### 3.6.3. Interferences

The influences of some potentially interferents on the detection of TBBPA were examined under the optimum conditions, and the result were shown in Table 2. It was found that 500-fold concentration of SO_4_^2−^, NO_3_^−^, CO_3_^2−^, Cl^−^, K^+^, Cu^2+^, Zn^2+^, Ca^2+^, Mg^2+^, Al^3+^; 200-fold of Fe^3+^; 5-fold of resorcinol, hydroquinone, BPAF; 2-fold of phenol, nonyl phenol, octyl phenol, 4-nitrophenol, 3-aminophenol, TBBPS, TBBPA-DAE, TBBPA-BHE, BPF, and equal amount of BPA could only change peak current less than 5%. It indicated the selectivity of the sensor was acceptable.

#### 3.6.4. Reproducibility and Stability

The reproducibility and storage stability of the SB3-16/ABPE were investigated to testify the precision and practicability of the proposed method by cyclic voltammetry. The relative standard deviation (RSD) of the present sensor responded to 0.5 µM TBBPA was calculated to be 4.2% among five measurements in the same sensor, revealing a favorable repeatability of SB3-16/ABPE. Eight modified electrodes were fabricated independently, and the RSD was 3.4% for the detection of 0.5 µM TBBPA respectively, showing a good reproducibility for constructing of SB3-16/ABPE. The stability of the sensor was also studied by detecting the peak current of 0.5 µM TBBPA. Prior to corresponding measurements, the modified electrode was kept at 4 °C in a refrigerator for 20 days. The electrode maintains most of its current response of 92%, indicating the reliable stability of the sensor.

#### 3.6.5. Practical Application

The fabricated electrode was introduced to determine TBBPA in pond water to testify its practical application. The sample was prepared as described in Section 2.4. For the reason that TBBPA was not detected from water samples, the recovery of this sensor was investigated by standard addition method in our study, and the results were displayed in Table 3. The recoveries of the sensor were from 92.4% to 100.6%. To investigate the accuracy of this approach, a conventional method of High-performance liquid chromatography (HPLC) was also employed. The results were consistent with the results acquired from HPLC, suggesting the reliable accuracy of the present method in practical applications.

## 4. Conclusions

In conclusion, a sensitive and simplified electrochemical sensor was constructed for the determination of TBBPA. The as-prepared sensor exhibited high accumulation efficiency to TBBPA, and greatly increased oxidation peak current of TBBPA due to the superior electrochemical properties of AB and good adsorption capacity of SB3-16. This sensor had many strengths including low cost, simple preparation process, good stability, excellent repeatability, and satisfactory sensitivity. The present sensor was also applied for the determination of TBBPA in water samples, with satisfactory recoveries from 92.4% to 100.6%, and the results were consistent with those of HPLC. Therefore, we hope the fabricated electrode can facilitate the determination of TBBPA and contribute to the control and management of TBBPA pollution.

## Figures and Tables

**Table 1:** Comparisons of analytical performance of various TBBPA sensors.

Sensors	Linear Range (µM)	Detection Limit (nM)	Reference
DODMA/GCE ^a^	0.0018–0.92	1.05	[21]
CTAB/NG-TPA ^b^/GCE	0.01–1	9	[22]
CTAB/CPE	0.0025–0.8	0.99	[39]
MMIP ^c^/CPE	0.005–2	0.77	[40]
SB3-16/ABPE	0.001–1	0.4	This work

^a^ DODMA/GCE: dioctadecyldimethylammonium bromide/glass carbon electrode; ^b^ NG-TPA: nitrogen-doped graphene-1, 3, 6, 8-pyrenetetrasulfonic acid; ^c^ MMIP: magnetic molecularly imprinted polymers.

**Table 2:** Interferences of substances on the oxidation peak current of 0.5 μM TBBPA.

Interferences	Concentrations (mM)	Signal Change (%)
SO_4_^2−^	0.25	−2.2
NO_3_^−^	0.25	+3.9
CO_3_^2−^	0.25	+4.7
Cl^−^	0.25	−3.2
K^+^	0.25	−2.0
Cu^2+^	0.25	+3.3
Zn^2+^	0.25	+2.5
Ca^2+^	0.25	−4.2
Mg^2+^	0.25	−3.8
Al^3+^	0.25	+4.8
Fe^3+^	0.1	+3.6
Resorcinol	0.0025	+4.5
Hydroquinone	0.0025	−2.9
BPAF	0.0025	−2.3
Phenol	0.001	+2.2
Nonyl phenol	0.001	+3.1
Octyl phenol	0.001	+2.1
4-Nitrophenol	0.001	+2.4
3-Aminophenol	0.001	+1.2
TBBPS	0.001	−1.5
TBBPA-DAE	0.001	−2.1
TBBPA-BHE	0.001	−4.2
BPF	0.001	+1.1
BPA	0.0005	+4.7

**Table 3:** Determination of TBBPA in water sample.

Samples	Added (µM)	This Method			HPLC		
		Found (µm)	Recovery (%)	Rsd ^a^ (%)	Found (µM)	Recovery (%)	RSD ^a^ (%)
1	0.05	0.0503	100.6	4.3	0.0478	95.6	2.6
2	0.10	0.0947	94.7	2.8	0.1023	102.3	2.1
3	0.50	0.462	92.4	3.5	0.492	98.4	1.7

^a^ Relative standard deviation for *n* = 3.
